# Generation of Mouse Spermatogonial Stem-Cell-Colonies in A Non-Adherent Culture

**DOI:** 10.22074/cellj.2016.4184

**Published:** 2017-02-22

**Authors:** Hossein Azizi, Thomas Skutella, Abdolhossein Shahverdi

**Affiliations:** 1 Faculty of Biotechnology, Amol University of Special Modern Technologies, Amol, Iran; 2 Institute for Anatomy and Cell Biology, Medical Faculty, University of Heidelberg, Heidelberg, Germany; 3 Department of Stem Cells and Developmental Biology, Cell Science Research Center, Royan Institute for Stem Cell Biology and Technology, ACECR, Tehran, Iran

**Keywords:** Spermatogonial Stem Cells, Cultivation, Proliferation

## Abstract

**Objective:**

The properties of self-renewal and division in spermatogonial stem cells (SSCs) support spermatogenesis. There is a number of reported methods for *in vitro* SSC culture systems. The development of a culture system that effectively supports isolation and selfrenewal of germline stem cells (GSCs) is of tremendous benefit for clinical trials, experimental research, and as potential treatment for male infertility. The current study aims to consider the cultivation and behavior of GSCs in a non-adherent culture system.

**Materials and Methods:**

In this experimental study, we cultured testicular cells from neonatal mice in agarose coated plates in the presence of Dulbecco’s modified Eagle’s medium (DMEM) medium (CTRL group), 10% fetal bovine serum (FBS)+DMEM (10% group), and growth factor (G group) that contained 2% FBS, glial cell-derived neurotrophic factor (GDNF), epidermal growth factor (EGF), and fibroblast growth factor (FGF). Mouse spermatogonial stem-like colonies were isolated approximately 3 weeks after digestion of the testis tissue. After passages 2-3, the identity of the mouse spermatogonial stem-like cells was confirmed by immunocytochemistry, reverse transcription-polymerase chain reaction (RT-PCR), and flow cytometry against the germ cell markers *α6, β1, c-Kit, Thy-1, c-Ret, Plzf,* and *Oct4*. The statistical significance between mean values in different groups was determined by one-way analysis of variance (ANOVA).

**Results:**

We observed spermatogonial stem-like colonies in the G and 10% groups, but not the CTRL group. Immunocytochemistry, flow cytometry, and RT-PCR confirmed expressions of germ cell markers in these cells. In the spermatogonial stem-like cells, we observed a significant expression (P<0.05) of germ cell markers in the G and 10% groups versus the testis cells (T). Their proliferative and apoptotic activities were examined by Ki67 and PI/annexin V-FITC. Alkaline phosphatase assay showed that mouse spermato- gonial stem-like colonies were partially positive.

**Conclusion:**

A non-adherent culture system could provide a favorable method for *in vitro* short-term culture of spermatogonial stem-like cell colonies.

## Introduction

The capability for self-renewal and differentiation enables spermatogonial stem cells (SSCs) to maintain spermatogenesis. During *in vitro* culture, SSCs can convert to pluripotent stem cells (1). Several research groups have reported SSC isolation and adherent culture. Useful methods to enrich SSCs include the use of extracellular matrices (ECM) such as laminin and collagen (2,5). It has been confirmed, that SSCs express α6- and β1- Integrin surface markers that bind to laminin (6). In addition, we employed fluorescence-activated cell sorting (FACS) or magnetic-activated cell sorting (MACS) against a number of different surface markers of α6 (CD49) and β1 (CD29) integrins (6, 7), CD9 (8), E-cadherin (9, 10), THY-1 (CD90) (11), and GFRa1 (12, 13), which are expressed on the cell surface of SSCs. Finally, a morphology-based selection of SSCs after the cultivation of total testicular cells on gelatincoated dishes (14-19) may be more valuable in comparison to other methods and due to the typical cellular morphology of SSCs (aligned or chain). The feeder layer is considered one of the main factors for growing SSCs. Different feeder layers enable researchers to observe diverse effects in the maintenance of SSCs. Mouse embryonic fibroblast (MEF) feeders are currently used in most SSC cultivations (20, 21). Similarly, testicular feeders that contain CD34 positive cells (22), SIM mouse embryo-derived thioguanine and ouabain-resistant fibroblasts (STO), or Sertoli cells (23, 24) at the feeder cell line can support SSC proliferation (25, 26).

While TM4 or SF7 somatic Sertoli cell lines reduced *in vitro* maintenance and the stem cell numbers of mouse male germline stem cells (GSCs) (27), it has been demonstrated that Sertoli cells can support the short-term cultivation of SSCs (23, 26). Unlike ST2 and PA6 bone marrow stromal cell lines, the OP9 bone marrow stromal cell line positively affected SSC maintenance (27). The extracellular nanofibrillar matrix could also support the maintenance of mouse neonate SSCs during short-term cultivation (28, 29). In addition, cultivatable SSCs in the feederfree culture could expand under serum-free conditions or without feeder cells on a laminincoated plate, however they did not expand in the absence of both serum- and feeder cells (3, 30). According to research, the germline potential decreased under serum- and feederfree culture conditions as determined by a lower SSC frequency after germline transplantation (31). Soluble growth factors could play a crucial role during the cultivation of SSCs, whereas the combination of growth factors, such as the glial cell-derived neurotrophic factor (GDNF), epidermal growth factor (EGF), and the basic fibroblast growth factor (bFGF) maintained SSCs in an undifferentiated state (32).

Suspension culture of embryonic stem cells has been reported. This culture system can support expansion, self-renewal, and pluripotency of pluripotent stem cells without their differentiation into embryoid bodies (33, 34). Floating aggregates in suspension culture express pluripotency markers and have the capability to differentiate into progeny of the three germ layers, both *in vitro* and *in vivo* (33). Larijani et al. (35) expanded pluripotent human embryonic stem cells (hESCs) and human-induced pluripotent stem cells (hiPSCs) in suspension aggregates by a simple, inexpensive and micro-carrier-free method. Similarly, according to research, a suspension culture of hESCs in the mTeSR medium is possible (36). However, as mentioned above, and although many studies have shown the *in vitro* culture of SSCs during an adherent culture system, limitations exist in terms of the maintenance of SSC self-renewal (37). In order to overcome this obstacle, the suspension culture system, which is known to have numerous advantages over adherent culture, has been used to cultivate germ cells (38, 39).

In the current study, we cultured digested testicular cells in a non-adherent culture plate coated with agarose in order to determine if neonatal testis germ cells had the capability to develop in a suspension culture.

## Materials and Methods

### Isolation of mouse spermatogonial stem-like colonies

Royan Institutional Review Board and Institutional Ethical Committee (Tehran, Iran) approved the animal experiments. Male mouse pups (5-7 days old, NMRI mouse) were purchased from Pasteur Institute (Iran). Mice testes were collected in phosphate buffered saline (PBS, Invitrogen, USA). After decapsulation, the testes seminiferous tubules were minced into slight pieces in Dulbecco’s modified Eagle’s medium (DMEM, Invitrogen, USA). We used a one-step enzymatic digestion protocol to obtain a single cell suspension. In brief, dissociated testicular tissue was placed in a digestion solution that contained collagenase IV (0.8 mg/ml), DNAse (0.5 mg/ml) and dispase (0.8 mg/ml) in HBSS buffer with Ca^2+^ and Mg^2+^ (PAA, USA) at 37˚C for 10 minutes (Table 1). All enzymes were purchased from Sigma-Aldrich. Digestion enzymes were halted with 10% fetal bovine serum (FBS), and the solution was pipetted to obtain a single cell suspension. After centrifugation, the specimens were washed with DMEM/F12, passed through a 70 μm nylon filter and centrifuged for 10 minutes at 1500 rpm. The supernatant was removed and approximately 1×10^6^ testicular cells were placed onto 10 cm^2^ tissue culture plates overlaid with 1% agarose. Cell viability was determined by the trypan blue exclusion assay. We divided the testicular cells into three groups for culture: i. Control (CTRL group) contained DMEM medium, ii. 10% FBS (10% group) contained DMEM medium+10% FBS, and iii. Growth factor (G group) that contained 2% FBS, GDNF (40 ng/ml), EGF (20 ng/ml), and FGF (20 ng/ml). The isolated testicular cells were maintained at 37˚C in an atmosphere of 5% CO_2_ in air for 21 days. The culture medium was changed every third day.

### Freezing and thawing of spermatogonial stem cells

The spermatogonial stem-like colonies were frozen in a cell freezing medium that consisted of 30% DMEM, 60% FBS and 10% dimethyl sulfoxide (DMSO). The cell pellets in the tube were re-suspended with a small volume of rest culture medium by gentle shaking. Then, 0.5-1 ml of associated freezing medium were added to each vial, followed by a quick transfer of the vials into an isopropanol freezing container, which was placed into a -80˚C freezer. After 24 hours, the frozen vials were transferred to a liquid nitrogen tank. The cells were thawed after transfer to a prewarmed DMEM medium, centrifuged and placed in culture medium.

### Immunofluorescence staining and alkaline phosphatase analysis

The immunostaining was performed in a 24-well plate by direct attachment or after a single cell of spermatogonial stem-like colonies. The cultured cells were fixed with 4% paraformaldehyde (Sigma- Aldrich, USA), then rinsed with PBS (Gibco, USA) and Tween20 (Sigma-Aldrich, USA). The cells were permeabilized by 0.2% Triton/PBS and blocked with 1% bovine serum albumin (BSA)/PBS. After removing the blocking solution, samples were incubated overnight with primary antibodies. After rinsing, the process was followed by incubation with species-specific secondary antibodies labeled with fluorescein isothiocyanate (FITC) fluorochrome (Table 1). Labeled cells were counterstained with 0.2 μg/ml 4´, 6-diamidino-2-phenylindole (DAPI) for 3 minutes at room temperature and fixed with Mowiol® 4-88 reagent. Negative controls for all markers consisted of the samples without any primary antibody. A fluorescence microscope (Olympus, BX51, Japan) was utilized for the examination of labeled cells, and their depictions displayed by an Olympus D70 camera. The alkaline phosphatase assay was performed using a commercial kit (Sigma- Aldrich, USA) as specified by the manufacturer.

### Flow cytometric analysis

PBS supplemented with 2% fetal calf serum (FCS) served as a staining buffer for the implementation of the flow cytometric reactions. After analysis of cell viability by trypan blue dye exclusion, the cells were washed twice in staining buffer, fixed in 4% paraformaldehyde, and permeabilized in 0.5% Triton X-100 (Darmstadt, Germany). The nonspecific antibody binding was blocked by a combination of 10% heat-inactivated goat serum in staining solution buffer. Approximately 1-1.5×10^5^ cells per sample were utilized. The incubation of the cells was performed with suitable amounts of primary antibodies or isotype-matched controls (Dako, X0927, 1:100). The samples were placed in staining buffer and incubated for 30 minutes at 4˚C with species-specific amounts of secondary antibodies. We conducted flow cytometric analysis with a BD-FACS Calibur Flow Cytometer system after the spermatogonial stem-like cells were washed. All experiments were conducted in triplicate and data were subsequently analyzed with WinMDI (2.9) software.

### Apoptosis assay

We utilized combined staining of FITCconjugated annexin V and propidium iodide (PI, IQP-116F) in order to examine for the presence of apoptosis and live spermatogonial stem-like cells. The harvested cells were washed with Ca2+ binding buffer [10 mM HEPES (pH=7.4), 140 mM NaCl, and 2.5 mM CaCl_2_], then re-suspended in 100 μL of the same solution buffer that contained FITC-conjugated annexin V. After a 20-minute incubation in the dark at 4˚C, the cells were diluted with 400 μL of binding solution buffer. The final step was followed by the addition of PI prior to flow cytometric analysis.

**Table 1:** List of materials


Materials	Company	Cat. no	Store

Anti mouse-Integrin α6 (CD49)	R&D	MAB 13501	-20
Anti mouse-Integrin β1 (CD29)	R&D	MAB 2405	-20
Anti mouse-c-Kit (CD117)	R&D	MAB1356	-20
Rabbit polyclonal c-Kit	Abcam	Ab16832	4
Rat monoclonal Thy-1 (CD90)	Abcam	Ab3105	4
Anti mouse monoclonal Plzf	Santa cruze	Sc-28319	4
Rabbit polyclonal Plzf	Abcam	Ab38739	-20
Rabbit polyclonal Ki67	Abcam	Ab15580	-20
Anti mouse Oct-4	Santa cruze	Sc-5279	4
Anti rabbit Oct-4	Cell signaling	C30A3	-20
Goat anti Rat IgG-FITC	Sigma	F6258	-20
Goat anti rabbit IgG Texas Red	Jackson	315075003	-20
Anti rabbit IgG HRP	Santa cruze	Sc-2301	4
Anti mouse IgG FITC	Sigma	F9006	-20
Goat anti rabbit IgG FITC	Abcam	Ab6717	-20
Sheep anti rabbit IgG Texas red	Abcam	Ab6793	-20
Goat anti rabbit IgG cy5	Abcam	Ab6564	-20
Rabbit anti mouse IgG Texas Red	Jackson	315-075-003	-20
Anti mouse IgM	Sigma	F9259	-20
GDNF	Sigma	G1777	-20
bFGF	Sigma	F0291	-20
rmEGF	R&D	2028-EG	-20
DNASE I	Roche	10104159001	4
Collagenase IV	Gibco	17104-019	4
Dispase	Gibco	17105-041	4
PI	Fluka	81845	4
collagenase	Sigma	C0130	-20
collagenase	Sigma	C1889	4


### RNA extraction and reverse transcriptionpolymerase chain reaction

For reverse transcription-polymerase chain reaction (RT-PCR), the total RNA was extracted from the testes and cells cultured using the NucleoSpin® RNA II kit (Macherey-Nagel, Düren, Germany). Prior to the RT step, RNA samples were purified with DNase I (EN0521, Fermentas, USA) to remove contaminating genomic DNA. cDNA synthesis was performed using 2 μg total RNA, oligo (dT)18, and a RevertAidTM H Minus First Strand cDNA Synthesis Kit (K1622, Fermentas) as specified by the manufacturer. The PCR reactions were performed in single PCR tubes and carried out using a Mastercycler gradient machine (Eppendorf, Germany). The cDNA samples were subjected to PCR amplification by mouse specific primers designed from different exons (Table 2). The reaction conditions for all primers were as follows: initial denaturation at 94˚C for 5 minutes followed by 30 cycles of denaturation at 94˚C for 30 seconds, annealing temperature at 59-70˚C for 45 seconds, extension time for 45 seconds at 72˚C, and a final polymerization at 72˚C for 10 minutes. The PCR products were examined by 1.5% agarose gel electrophoresis, stained with ethidium bromide (10 μg/ml), then visualized and photographed on a UV transilluminator (UVIdoc, UK).

**Table 2:** List of primers


Name	Primer sequence (5ˊ-3ˊ)	Product size	Annealing TM (˚C)

α6-Intergrin	F: CTC AGA ATA TCA AGC TCC CTR: AAA CAC TAA TAG AGC CAG CA	148	60
β1-Integrin	F: GAC ATT ACT CAG ATC CAA CCAR: AGG TAG TAG AGA TCA ATA GGG T	115	60
c-kit/(CD117)	F: CTA AAG ATG AAC CCT CAG CCTR: GCA TAA CAC ATG AAC ACT CCA	142	60
Thy-1/CD90	F: CTC TCC TGC TCT CAG TCT TGR: AGT TAT CCT TGG TGT TAT TCT CAT	119	60
Nanog	F: CTG ATT CTT CTA CCA GTC CCAR: AAA CCA GGT CTT AAC CTG CTT AT	235	60
Klf4	F: ACG ATC GTC GCC CCG GAA AAG GAC CR: TGA TTG TGA TGC TTT CTG GCT GGG CTC C		
Sox2	F: GCT GGG AGA AAG AAG AGG AGR: ATC TGG CGG AGA ATA GTT GG	180	60
c-Myc	F: GCC TAC ATC CTG TCC ATT CAR: AAC CGT TCT CCT TAC TCT CA		
GAPDH	F: CAA CTC CCA CTC TTC CAC TTR: GCA GCG AAC TTT ATT GAT GGT A	319	60


TM; Melting temperature.

### Ultrastructure of spermatogonial stem-like cell colonies

Spermatogonial stem-like cell colonies grown in the G and 10% groups were washed twice with PBS, pre-fixed with 2.5% buffered glutaraldehyde in 0.1 M PBS for 2 hours, then post-fixed with 1% aqueous osmium tetroxide for 1.5 hours. After dehydration through an ascending ethanol series (30, 50, 70, 80, 90, and 100%), the samples were dried in an air-dryer, mounted on a stab, and gold-coated using a sputter coater (EM/TECH, K 350, England). The samples were observed by scanning electron microscope (VEGA\TESCAN, Czech Republic).

## Results

**Fig.1:**
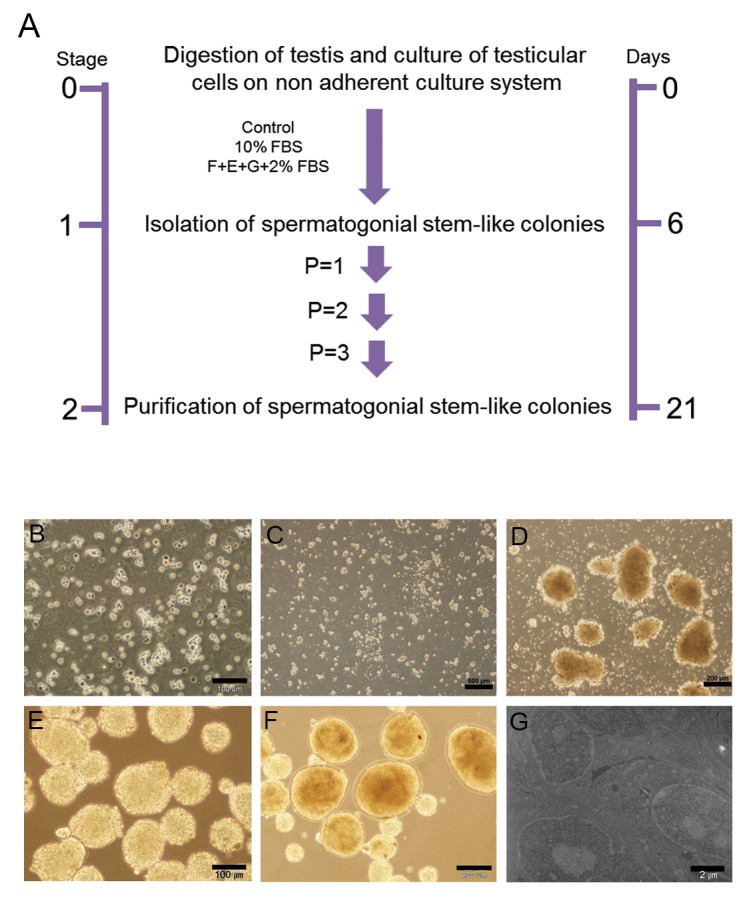
Generation of spermatogonial stem-like colonies. A. Protocol for the generation of spermatogonial stem-like colonies from mouse testis, B. Testis cells after: digestion, C. Culture on a non-adherent plate after 3 days, D. 7 days, E. Passage-3, F. Spermatogonial stem-like cell colonies form after cryopreservation, and G. Electron micrograph analysis for spermatogonial stem-like cell colonies. FBS; Fetal bovine serum.

We sought to determine if testicular cells could form GSC colonies in a non-adherent culture system. In this study, approximately 1×10^6^ testicular cells obtained from NMRI strain pups were cultured on 10 cm^2^ tissue culture plates overlaid with 1% agarose. In this protocol, testicular cells divided into three groups (CTRL, G and 10%) were cultured for 21 days (Fig .1A). We observed the formation of spermatogonial stem-like cell colonies in the G and 10% groups 7 days after cultivation in a non-adherent system (Fig .1B-D). In order to generate additional pure colonies and to decrease the amounts of single cells in the primary culture, we picked up spermatogonial stem-like cell colonies for further cultivation in new culture plates after trypsinization (Fig .1E). There were no spermatogonial stem-like cell colonies observed in the CTRL group; however, these cells reconfigured in the G and 10% groups after cryopreservation (Fig .1F). Electron micrograph analyses showed that spermatogonial stem-like cell colonies in the G and 10% groups had a similar morphology compared to SSCs *in vivo*, which localized on the basement membrane of seminiferous tubules and had a high nucleus/cytoplasm ratio (Fig .1G).

**Fig.2:**
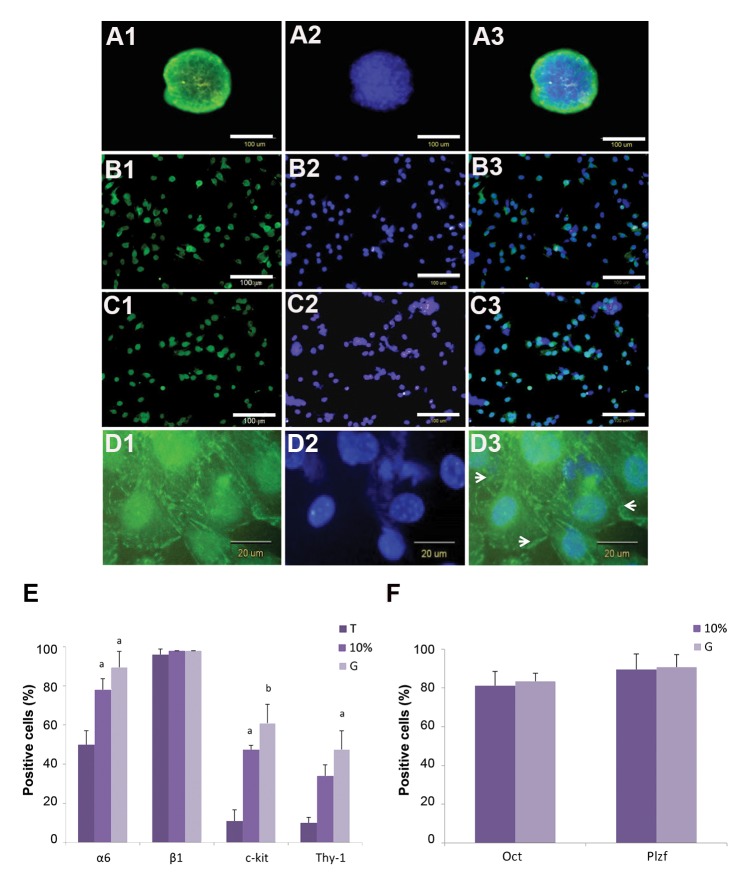
Characterization of spermatogonial stem-like colonies. Immunocytochemistry results showed that spermatogonial stem-like colonies expressed germ cell markers: A. α6-Integrin, B. Oct4, C. Plzf, D. c-Ret, E. Flow cytometry analyses for expression of surface markers: α6-Integrin, β1-Integrin, c-Kit, and Thy-1 in the testis (T), 10%, and G groups, and F. Flow cytometry analyses for expressions of Oct4 and Plzf transcription factor in the 10% and G groups. a; P<0.05 versus the T group and b; At least P<0.005 versus the T group.

Spermatogonial stem-like cells were characterized by immunocytochemistry assays 21 days after cultivation. Immunofluorescence staining proved that the colonies were positive for α6-Integrin, Plzf, Oct4, and c-Ret (Fig .2). Flow cytometry analysis of the cells 21 days after culture showed cells in isolated colonies that were positive for the surface markers α6-Integrin, β1-Integrin, c-Kit, and Thy-1 (Fig .2E). We observed significantly higher expressions of α6-Integrin, c-Kit, and Thy-1 in the G and 10% groups compared to the testis group (at least P<0.05). Similarly, the expressions of transcription factors Plzf and Oct4 resembled their expressions in the G and 10% groups (Fig .2F).

**Fig.3:**
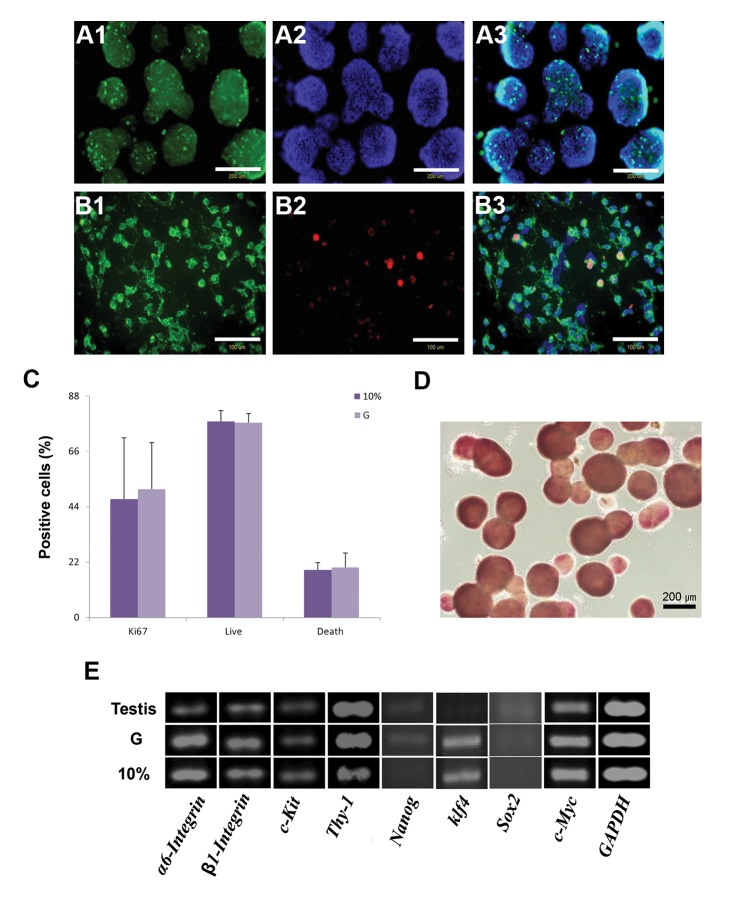
Characterization of spermatogonial stem-like colonies. A. Expression of Ki67 in the spermatogonial stem-like colonies, B. Double staining of Ki67 with β1-Integrin, C. Flow cytometry analysis for expression of Ki67 and also quantification for annexin V in isolated colonies from the G and 10% groups, D. Alkaline phosphatase assay for spermatogonial stem-like colonies in the G group, and E. mRNA expression of germ cell genes: *α6-Integrin, β1-Integrin, c-Kit, Thy-1, Nanog, Klf4, Sox2,* and *c-Myc* in the G, 10%, and testis groups.

We sought to determine if spermatogonial stem-like cells in the non-adherent culture could play a role in the proliferation of cells in the colonies by conducting the Ki67 cell proliferation assay at 21 days after cultivation (Fig .3A). Immunofluorescence staining showed that colonies positive for Ki67 co-stained with β1-Integrin (Fig .3B). Flow cytometry analysis has confirmed the expression of Ki67 in the cells from isolated colonies in the G and 10% groups. Ki67 is a nuclear non-histone protein expressed during cell proliferation (40). A flow cytometry quantification for cells stained with annexin V indicated a large number of surviving cells and a few apoptotic cells in the G and 10% groups (Fig .3C). The alkaline phosphatase assay for spermatogonial stem-like colonies in the G and 10% groups showed alkaline phosphatase expression after 21 days during suspension cultivation (Fig .3D). We also evaluated the mRNA expression of germ cell genes *α6-Integrin, β1-Integrin, c-Kit, Thy-1, Nanog, Klf4, Sox2,* and *c-Myc* on isolated spermatogonial stem-like cells in the G, 10%, and control groups. We observed that *α6-Integrin, β1-Integrin, c-Kit, Thy-1,* and *c-Myc* clearly expressed in all groups, whereas Klf4 expressed in the G and 10% groups, but not in testis cells. We did not observe or could only observe a very low expression of *Nanog* and *Sox2* in all groups (Fig .3E).

## Discussion

In this study, we reported the effect of a non- adherent culture system on mouse testicular cells. The isolated spermatogonial stem-like cell colonies in a suspension culture expressed germ cell markers, and featured proliferation and survival characteristics. These phenomena possibly evinced an ideal culture system for analyzing differences in the testes niche microenvironment. Several reports demonstrated the beneficial influence of suspension culture for embryonic stem cells (33,35,41). We cultured mouse neonate testicular cells on tissue cultures coated with agarose in order to provide a non-adherent surface and to avoid the adhesion of testicular cells. As mentioned earlier, spermatogonial stem-like colonies did not form in the control group that lacked growth factors. The growth factors GDNF and either FGF2 or EGF have been shown to be essential for self-renewal, expansion, and differentiation of SSCs (21,42). In our experiment, we did not observe any obvious differences between the 10% FBS and G groups during the short-term culture period. It seemed that spermatogonial stem-like colonies, which enriched in both groups, had the same germ cell gene expression patterns. Recently, researchers used suspension bioreactors for the enrichment of testicular germ cells (38,39). 

While it has been demonstrated that a low concentration of serum was beneficial for the short-term culture of goat SSCs Bahadorani et al. (43) showed that the long-term culture of SSCs depended on an slight increments of serum concentration. Although a high concentration of serum in SSCs culture has been demonstrated (21,23,27), Kanatsu-Shinohara et al. (20) presented a defined medium with growth factors and low percentage of FBS for short- and long-term SSC cultivation. We demonstrated that isolated spermatogonial stem-like cell colonies in both groups clearly expressed germ cell markers, which confirmed previous reports (44,45). The GSCs expressed some transcription factors (*Pou5f1, Sox2, c-Myc,* and *Klf4*) usually required for reprogramming (46). In the spermatogonial stem-like colonies, we have observed a low expression of *Nanog*, which might be the cause of PTEN and TRP53 suppression (47). Plzf plays an important role in maintenance and proliferation of SSCs (48,49). 

We also observed that colonies in a non-adherent culture exhibited strong survival and proliferative characteristics (45,50). It has been demonstrated by activation of specific signaling pathways that several factors are essential for the survival of cultured SSCs (51,53). 

## Conclusion

These results may prove that spermatogonial stem-like cell colonies do not only form in a non-adherent culture system, but that this system also supports the maintenance of cells by affecting expressions of associated genes. Another advantage of this culture method for testicular cells is the ability to analyze different growth factors and associated signaling pathways that concern spermatogonial stem-like cell colonies without directly affecting the ECM. ECM interactions lead to signal transduction mechanisms in the cells, regulating their fate and behavior. Therefore, the application of our non-adherent culture system or its combination with an adherent culture may be useful for future applications of testicular cells in stem cell therapy or regenerative medicine. 
